# A Comparative Analysis of Extra-Embryonic Endoderm Cell Lines

**DOI:** 10.1371/journal.pone.0012016

**Published:** 2010-08-06

**Authors:** Kemar Brown, Stephanie Legros, Jérôme Artus, Michael Xavier Doss, Raya Khanin, Anna-Katerina Hadjantonakis, Ann Foley

**Affiliations:** 1 Greenberg Division of Cardiology, Weill Cornell Medical College, New York, New York, United States of America; 2 Developmental Biology Program, Sloan-Kettering Institute, New York, New York, United States of America; 3 Computational Biology Program, Sloan-Kettering Institute, New York, New York, United States of America; INSERM, France

## Abstract

Prior to gastrulation in the mouse, all endodermal cells arise from the primitive endoderm of the blastocyst stage embryo. Primitive endoderm and its derivatives are generally referred to as extra-embryonic endoderm (ExEn) because the majority of these cells contribute to extra-embryonic lineages encompassing the visceral endoderm (VE) and the parietal endoderm (PE). During gastrulation, the definitive endoderm (DE) forms by ingression of cells from the epiblast. The DE comprises most of the cells of the gut and its accessory organs. Despite their different origins and fates, there is a surprising amount of overlap in marker expression between the ExEn and DE, making it difficult to distinguish between these cell types by marker analysis. This is significant for two main reasons. First, because endodermal organs, such as the liver and pancreas, play important physiological roles in adult animals, much experimental effort has been directed in recent years toward the establishment of protocols for the efficient derivation of endodermal cell types *in vitro*. Conversely, factors secreted by the VE play pivotal roles that cannot be attributed to the DE in early axis formation, heart formation and the patterning of the anterior nervous system. Thus, efforts in both of these areas have been hampered by a lack of markers that clearly distinguish between ExEn and DE. To further understand the ExEn we have undertaken a comparative analysis of three ExEn-like cell lines (END2, PYS2 and XEN). PYS2 cells are derived from embryonal carcinomas (EC) of 129 strain mice and have been characterized as parietal endoderm-like [1], END2 cells are derived from P19 ECs and described as visceral endoderm-like, while XEN cells are derived from blastocyst stage embryos and are described as primitive endoderm-like. Our analysis suggests that none of these cell lines represent a *bona fide* single *in vivo* lineage. Both PYS2 and XEN cells represent mixed populations expressing markers for several ExEn lineages. Conversely END2 cells, which were previously characterized as VE-like, fail to express many markers that are widely expressed in the VE, but instead express markers for only a subset of the VE, the anterior visceral endoderm. In addition END2 cells also express markers for the PE. We extended these observations with microarray analysis which was used to probe and refine previously published data sets of genes proposed to distinguish between DE and VE. Finally, genome-wide pathway analysis revealed that SMAD-independent TGFbeta signaling through a TAK1/p38/JNK or TAK1/NLK pathway may represent one mode of intracellular signaling shared by all three of these lines, and suggests that factors downstream of these pathways may mediate some functions of the ExEn. These studies represent the first step in the development of XEN cells as a powerful molecular genetic tool to study the endodermal signals that mediate the important developmental functions of the extra-embryonic endoderm. Our data refine our current knowledge of markers that distinguish various subtypes of endoderm. In addition, pathway analysis suggests that the ExEn may mediate some of its functions through a non-classical MAP Kinase signaling pathway downstream of TAK1.

## Introduction

Studies in amphibians, avians and mice demonstrate that endodermal cells play both inductive roles and make important cellular contributions to organ formation. Endodermally derived organs such as the liver and pancreas serve important secretory functions that are required for homeostasis in the adult organism and because of this, much effort has been exerted in recent years toward the development of protocols for the directed differentiation of specific endodermal subtypes. Toward these efforts, the identification of secreted endodermal factors that mediate their inductive functions would also be highly desirable. However, these efforts have been hampered by a lack of markers that efficiently distinguish one type of endoderm from another. One possible reason for this is that endoderm constitutes only a small percentage of cells in the developing embryo, and consequently, slow progress has been made in the identification of regional specific markers within the endoderm. Furthermore, it has been noted that there is tremendous overlap in marker expression between the visceral extra-embryonic endoderm and the gut endoderm of the embryo. Recent efforts to characterize markers that distinguish these lineages have relied on endoderm derived from ES cell sources followed by FACS purification with the aid of antibodies that recognize different types of endoderm [2], [3]. While these approaches have identified multiple lineage restricted endodermal markers not all of the “hits” have been validated by further experimentation.

**Figure 1:**
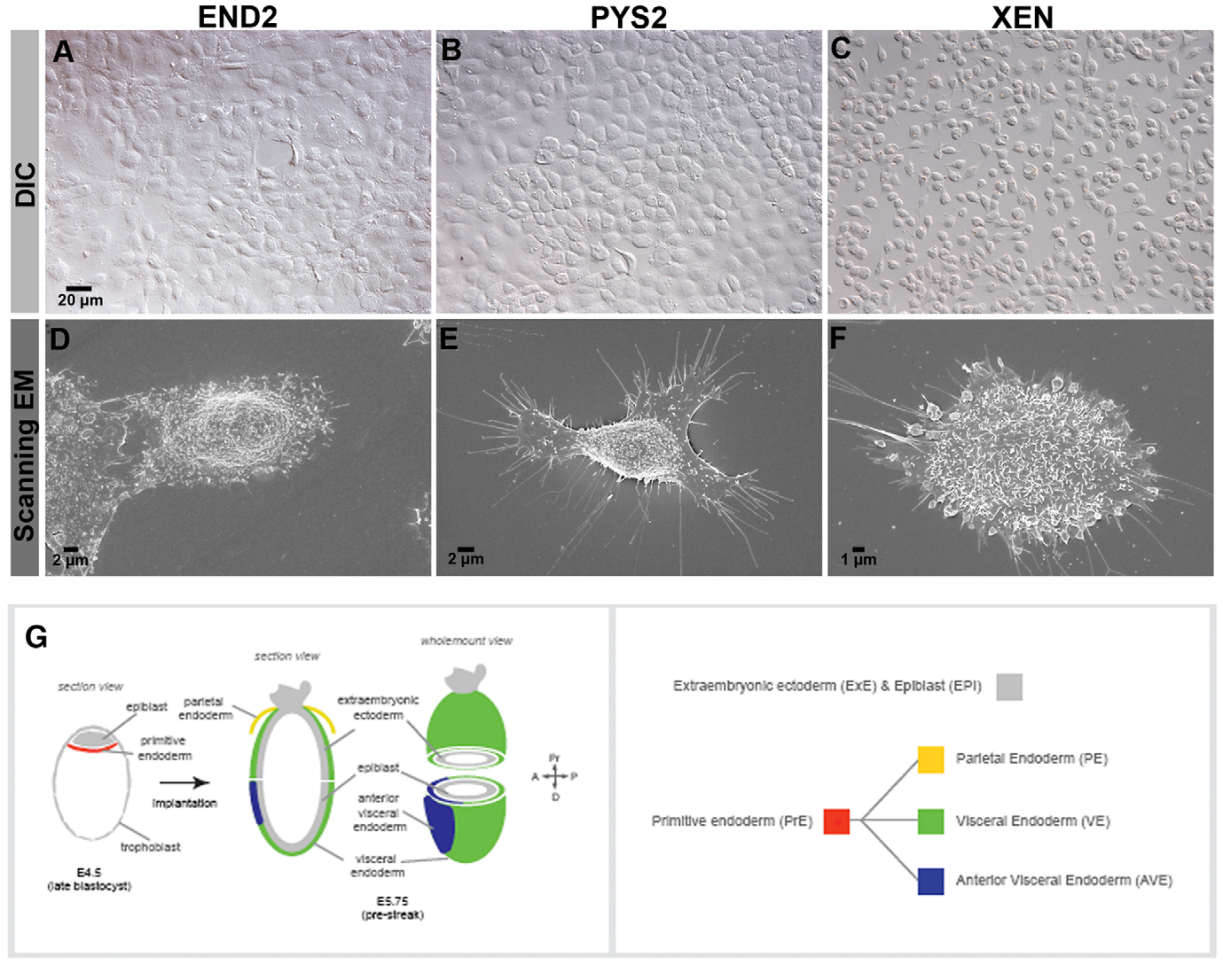
Morphological characterization of END2, PYS2 and XEN cells. DIC (**1** **A–C**) and Scanning EM (**1** **D–F**) images of END2 (**1** **A, D**), PYS2 (**1** **B, E**) and XEN (**1C, F**) cells reveal morphological details of the cell lines used in these studies. **G.** Cartoon depicting early endodermal lineages in the mouse embryo prior to gastrulation. The primitive endoderm (red) forms in the pre-implantation blastocyst stage embryo and subsequently expands and differentiates into parietal endoderm (yellow), visceral endoderm (green) and anterior visceral endoderm (blue). Visceral endoderm is also sub-divided into embryonic and extra-embryonic regions based both on location relative to the embryonic/extra-embryonic junction of the epiblast (grey), fate and marker expression.

In the mouse it has long been assumed that there are two distinct phases of endoderm formation such that extra-embryonic endoderm forms prior to gastrulation and is derived from the primitive endoderm (Fig. 1G, red), while the definitive endoderm rises from the epiblast during gastrulation. Prior to gastrulation, the original primitive endoderm expands with the growing embryo and becomes subdivided into PE (Fig. 1G, yellow) and VE (Fig. 1G, green) based on their position relative to the egg cylinder. The VE itself is further divided into sub-regions, including the anterior visceral endoderm (AVE) (Fig. 1G, blue). In this study, we characterize and compare three cell lines that are either derived from the primitive endoderm or have been reported to resemble these primitive endoderm-derived lineages. Three ExEn cells lines were examined in detail by immunocytochemistry, qRT-PCR and microarray analysis, using well-characterized markers for ExEn and definitive endoderm, and a more elaborate panel of putative ExEn markers, previously identified as distinguishing between different subtypes of endoderm. These studies confirm that each of these ExEn cell lines exhibits high molecular correlation to visceral and parietal endoderm and little or no similarity to definitive (epiblast-derived) endoderm. This comparative gene analysis also refines a growing list of markers that have been proposed to distinguish between VE and DE. By providing a clearer picture of endodermal subtypes, these studies should assist the development of experimental protocols that require a distinction between embryonic and extra-embryonic lineages.

Finally, consistent with our previous embryological studies, pathway analysis from microarray data reveals that molecules downstream of TGFbeta-family members are highly represented in these cell lines and suggests that both SMAD-dependent and SMAD-independent TGFbeta signaling could mediate the inductive function of these cell lines.

## Results

### Extra-embryonic endoderm stem cells (XEN cells) and PYS2 cells but not END2, express markers characteristic of the primitive endoderm

END2 and PYS2 cells have been described previously, based on cell morphology and marker expression [1], [4], [5], to be similar to visceral endoderm (VE) and parietal endoderm (PE), respectively. Because these cells were originally derived from EC cell lines, they may not represent true endodermal lineages but rather, endoderm-like populations. Recently described protocols allow for the isolation of ExEn stem cells (XEN cells) directly from blastocyst stage mouse embryos [6], and as such, are more likely to represent endogenous endodermal cell types. For this study, we derived a XEN cell line from wild type mouse blastocysts of the ICR strain. Although each of the three cell lines are relatively flat and exhibit a cobblestone appearance when confluent, bright field microscopy reveals that each of the three cell lines is morphologically distinct from the other two (Fig. 1A–C). Scanning electron microscopy of cells plated at low density reveals that all three cell types are rounded in appearance and densely covered with microvilli, with END2 and PYS2 cells forming large lamellipodia (Fig. 1D–F). These data demonstrate that, like END2 and PYS2 cells, XEN cells exhibit an endodermal morphology.

**Figure 2:**
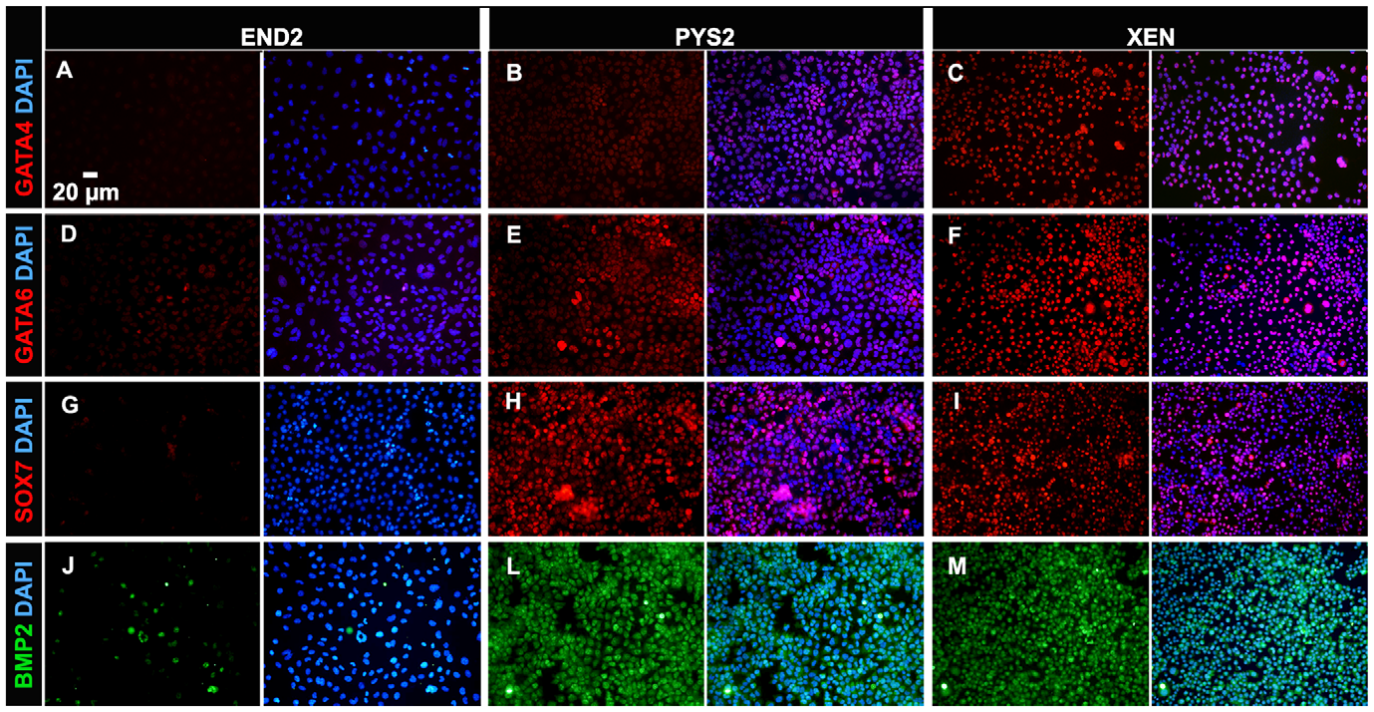
Immunocytochemical analysis of ExEn cell lines. Immunocytochemical analysis of confluent END2 (**A, D, G, J**), PYS2 (**B, E, H, L**) and XEN (**C, F, I, M**) cells showing the expression of GATA4 (**A, B, C**), GATA6 (**D, E, F**), SOX7 (**G, H, I**) and BMP2 (**J, L, M**) protein in END2, PYS2 and XEN cells. Merged images with DAPI staining (blue nuclei in all images) reveal ubiquitous expression of GATA4 and GATA6 in both PYS2 and XEN cells (**B, C, E, F**). SOX7 is ubiquitously expressed in PYS2 cells (**H**), while XEN cells express SOX7 only in a subset of cells (**I**). In END2 cells, GATA6 expression is limited to a small subset of cells (**D**) while GATA4 and SOX7 are not expressed (**A, G**). BMP2 is ubiquitously expressed by PYS2 and XEN cells (**L, M**), while END2 express BMP2 only in a subset of cells (**J**).

Each of these cell lines was assessed by immunocytochemistry, for a panel of markers characteristic of the primitive endoderm including SOX7, GATA4 and GATA6 [7], [8], [9], [10]. Both XEN and PYS2 cells are recognized by antibodies against GATA4, GATA6, and SOX7 (Fig. 2 B, C, E, F, H, I). However, only a subset of XEN cells express SOX7 (Fig. 2I). By contrast, END2 cells express only low levels of GATA6 (Fig. 2D) and do not express GATA4 or SOX7 (Fig. 2 A, G). In these studies, PYS2 cells showed the most uniform expression of these primitive endoderm markers. By contrast, END2 and XEN cells may represent mixed or fluctuating populations of primitive endoderm and other lineages since their expression of these markers was more heterogeneous. In particular, the failure of END2 cells to express GATA4 and SOX7, suggests that there are few if any primitive endoderm cells within this line. While they do exhibit heterogeneous expression of GATA6 it should be noted that this gene also marks other endodermal subtypes including the VE. The heterogeneity of END2 cells is also demonstrated by the expression of BMP2, which is proposed to be a major signaling molecule from the endoderm [11], [12], [13], [14], [15], [16], [17]. BMP2 is uniformly expressed in PYS2 and XEN cells (Fig. 2L, M) but only expressed in a small subset of END2 cells (Fig. 2J). Overall these data suggest that END2 cells represent a heterogeneous endodermal population with little resemblance to the primitive endoderm.

### Detailed marker analysis demonstrates that END2 cells are molecularly divergent from XEN and PYS2 cells

**Figure 3:**
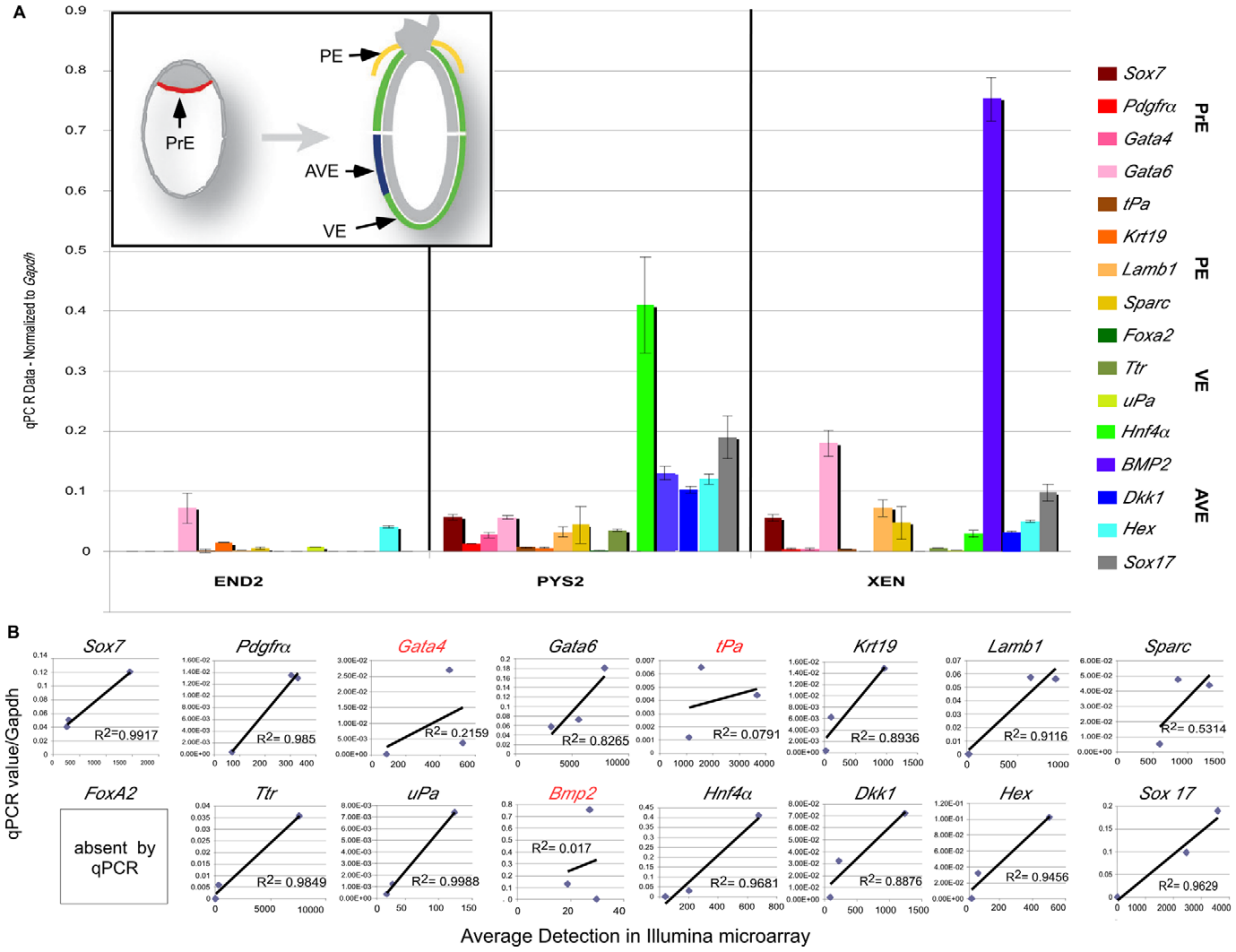
Heart inducing cell lines express markers characteristic of several primitive endoderm lineages. **A.** Summary of Real-Time PCR on END2, PYS2 and XEN cells. Insert, cartoon showing embryonic lineages assessed, primitive endoderm (reds), parietal endoderm (oranges/yellows), visceral endoderm (greens) and AVE (blues). The panel of markers assessed include markers for primitive endoderm (*Sox7*, *Pdgfra, Gata4, Gata6*), parietal endoderm (*tPA, Krt19, Lamb1* and *SPARC*), visceral endoderm, (*FoxA2*, *Ttr*, u*PA and HNF4a*), anterior visceral endoderm (*Dkk1, Cerl, Hex*), the regionally restricted VE marker *Bmp2* and the definitive/pan endoderm marker *Sox17*. **B.** Linear regression analysis comparing real-time PCR data to averaged fluorscence detection in the Illumina Microarray. 80% of markers that we compared showed strong correlation between the qRT-PCR data and microarray detection. The data discussed in this publication have been deposited in NCBI's Gene Expression Omnibus [78] and are accessible through GEO Series accession number GSE19564 (http://www.ncbi.nlm.nih.gov/geo/query/acc.cgi?acc=GSE1956).

To further characterize these cell lines, we used qRT-PCR (Fig. 3A), to examine a panel of markers representing several ExEn lineages (Fig. 1G and Fig. 3, insert) including PrE, PE, VE and anterior visceral endoderm (AVE). *Sox7* [7], *Pdgfra* [18], *Gata4* [10], and *Gata6* [19] are expressed in the primitive endoderm of the mouse blastocyst and are thought to be among the earliest ExEn markers. XEN and PYS2 cells express all of these markers, but END2 cells express only *Gata6*. Note that all of these markers are also expressed in derivatives of the primitive endoderm including the PE and VE and as a consequence, there are no known markers that are uniquely expressed in the primitive endoderm. All three cell lines express markers for the PE including *t-type Plasminogen activator* (*tPA*) [20], *Cytokeratin 19* (*Krt19*) [21], *Laminin B1* (*Lamb1*) [22] and *Sparc* [23], although END2 cells express these at relatively lower levels as compared to the other cell lines.

We next determined the transcriptional status of markers that are widely expressed throughout the VE including *FoxA2* [24], *Transthyretin (Ttr*) [25] *u-type Plasminogen activator* (*uPA*) [26] and *Hepatocyte nuclear factor 4, (Hnf4a)* [24]. *Ttr* and *uPA* are expressed by all three of these cell lines (although at lower levels in END2). *Hnf4a* is expressed by PYS2 and XEN cells, but not by END2. *FoxA2* is only expressed by PYS2 cells. None of these cell lines express *Vilin* (data not shown).

We next assessed markers whose expression is restricted (either spatially or temporally) within the VE. The extra-embryonic VE that lies proximally over the extra-embryonic ectoderm of the mouse embryo (Fig. 1G) expresses *Sox7*, *Sox17* and also upregulates *alpha fetoprotein* (*Afp*) after gastrulation has been initiated. Prior to gastrulation, *Afp* and *Sox17* also mark the distally positioned VE that overlies the epiblast (the embryonic VE) (Fig. 1G and 3, insert) [27]. Of these, *Sox7* and *Sox17* are present in both PYS2 and XEN but not END2 cells. *Afp* is only expressed in PYS2 cells. Thus, PYS2 and XEN cells express markers for both the extra-embryonic VE and the embryonic VE, whereas END2 cells only expressed panVE markers such as *Ttr*.

Finally, we assessed a panel of markers that are spatially restricted in the VE. *Dkk-1* [28], *Cerl* [29], [30], [31], and *Hex* [32] are all known to be expressed in the AVE of the mouse embryo. As has previously been shown for XEN cells [6], all three ExEn cell lines express *Hex*. PYS2 and XEN cells also express *Dkk1*. None of the cell lines express *Cerl* (data not shown). Consistent with our immunocytochemical analysis, BMP2, which is also spatially restricted within the VE, is expressed by all three of the ExEn cell lines.

Both the patchy expression of these markers when assessed by immunoctyochemistry and the relatively lower expression of mRNAs for these genes when assessed by qRT-PCR, are consistent with the idea that END2 cells are a heterogeneous population in which a small subset of cells express markers for the VE (or more likely, a subtype of VE), whereas PYS2 and XEN cells are more homogeneous and express a broad array of markers for the primitive endoderm and its derivatives.

To further analyze these cell lines, we performed a comparative microarray analysis. To confirm the consistency between the array data and data collected from qRT-PCR and immunocytochemistry analyses, we examined the same panel of markers initially assessed by qRT-PCR. Averaged fluorescent detection for each marker was plotted versus qRT-PCR data normalized to *Gapdh* and R^2^ values were determined from the line of best-fit. Importantly, a high degree of correlation is found between the two data sets, and over 80% of the genes tested had R^2^ values close to one (Fig. 3B). We found only three notable exceptions. First, *tPA* showed the same basic trend between qRT-PCR and microarray, but with low numerical correlation. This could reflect non-specific amplification by qRT-PCR or a problem with the array probe. In addition, a single probe for *Gata4* is highly recognized in the array by END2 cells. *Gata4*, however, is absent in END2 cells by both qRT-PCR (Fig. 3A) and immunocytochemistry (Fig. 1A). Finally, *Bmp2* is not detected in the array but is highly expressed by PYS2 and XEN cell lines, as assessed by qRT-PCR and immunocytochemistry (Fig. 2 J–M and Fig. 3A). This suggests that these particular probes may either recognize non-specific transcripts or splice variants of the target genes and highlights the need for independent verification of candidates identified by probing microarrays. Overall, there is significant agreement between microarray data and our other assays.

**Figure 4:**
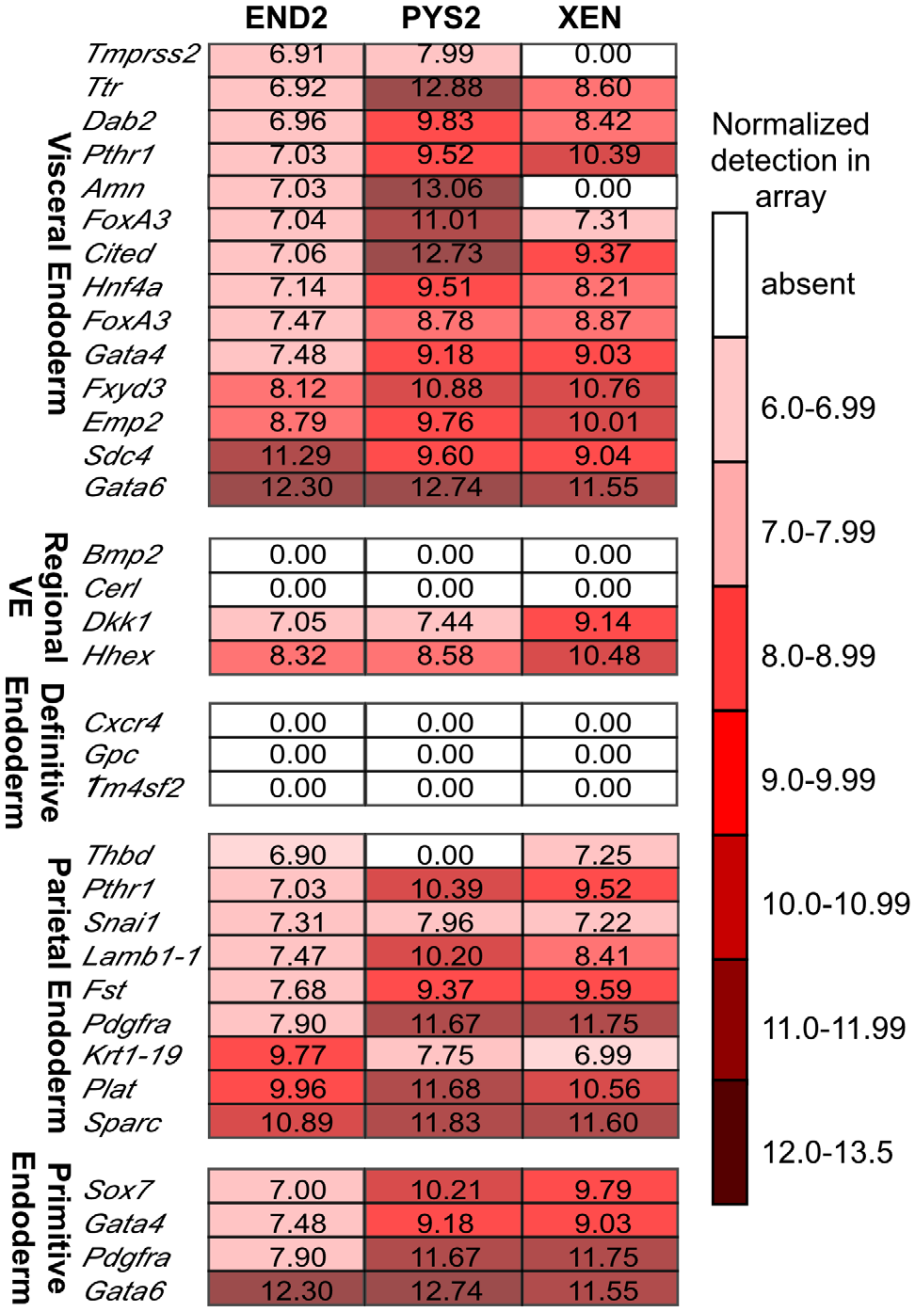
Heat map analysis of well-characterized markers for different endodermal cell types. Illumina microarray data for genes that are expressed in various endoderm subtypes (depicted as heat maps) includes regionally restricted markers representing the VE, DE, PE and PrE. Each of the three cell lines expresses markers for VE, PE and PrE. None of the cell lines express markers that are diagnostic for DE. Fluorescence data was indicated as 0.00 if the p-value of detection was greater than 0.01.

Having confirmed a high degree of correlation between qRT-PCR data and the array, a large-scale analysis of markers for endodermal cell types was undertaken using microarray data (Fig. 4). Studies using immunohistochemistry and *in situ* hybridization studies have identified a relatively small number of genes that are differentially expressed in the endoderm. Indeed, many of these markers are expressed in more than one endodermal subtype. For example, the primitive endoderm markers used in this study (*Gata4, Gata6, Sox7* and *Pdgfr*α) are also expressed in other endodermal subtypes. Thus few truly diagnostic markers that distinguish endodermal subtypes have been identified. Two recent studies aimed at identifying markers that distinguish between VE and DE were based largely on comparative analysis of endodermal cell populations sorted by expression of endodermal markers. First, Sherwood *et al*. used differential expression of the epitopes for *EpCAM*, *Dba* and *Ssea-4* [2]. Later, Yasunga et al. used differential expression of *Goosecoid* and *Sox17* [3]. Here we sought to analyze these data sets by comparative analysis of the three ExEn cell lines. First, we analyzed our array data based on well-characterized markers for endodermal subsets (Fig. 4). As expected all three of these cell lines express most of the VE specific markers. Notably, all three express high levels of *Fxyd3, Emp2, Sdc4* and *Gata6*, suggesting that these markers, in particular, are highly diagnostic for the VE fate. By contrast, a second subset of VE markers including *Ttr, Dab2, Pthr1* and *Cited* are highly expressed by PYS2 and XEN cells but not by END2 cells, suggesting that they mark a specific subset of cells within the VE. In addition, all three cell lines express the AVE markers *Hex* and *Dkk1* but not *Cerl*.

None of the cell lines express markers reported to be diagnostic for DE including *Cxcr4, Gpc1* and *Tm4sf2* [3]. This would seem to confirm that these cell lines do not possess characteristics of the DE and conversely support the notion that these markers are diagnostic for DE but not ExEn cell types.

We further analyzed these cells for PE markers and found, as expected based on our previous analysis, that PYS2 and XEN but not END2 cells show high expression of PE markers. Finally, in confirmation of our previous findings, XEN cells and PYS2 cells but not END2 cells express high levels of markers for the primitive endoderm. In addition, each cell had a specific subset of uniquely expressed markers that are either higher or lower as compared to the other two cell lines. Since these cell lines have in other assays been shown to have inductive effects, such as activating heart formation [4], [5], [33], [34], [35], these differences might be exploited to identify specific inductive signals within the individual cell lines.

**Figure 5:**
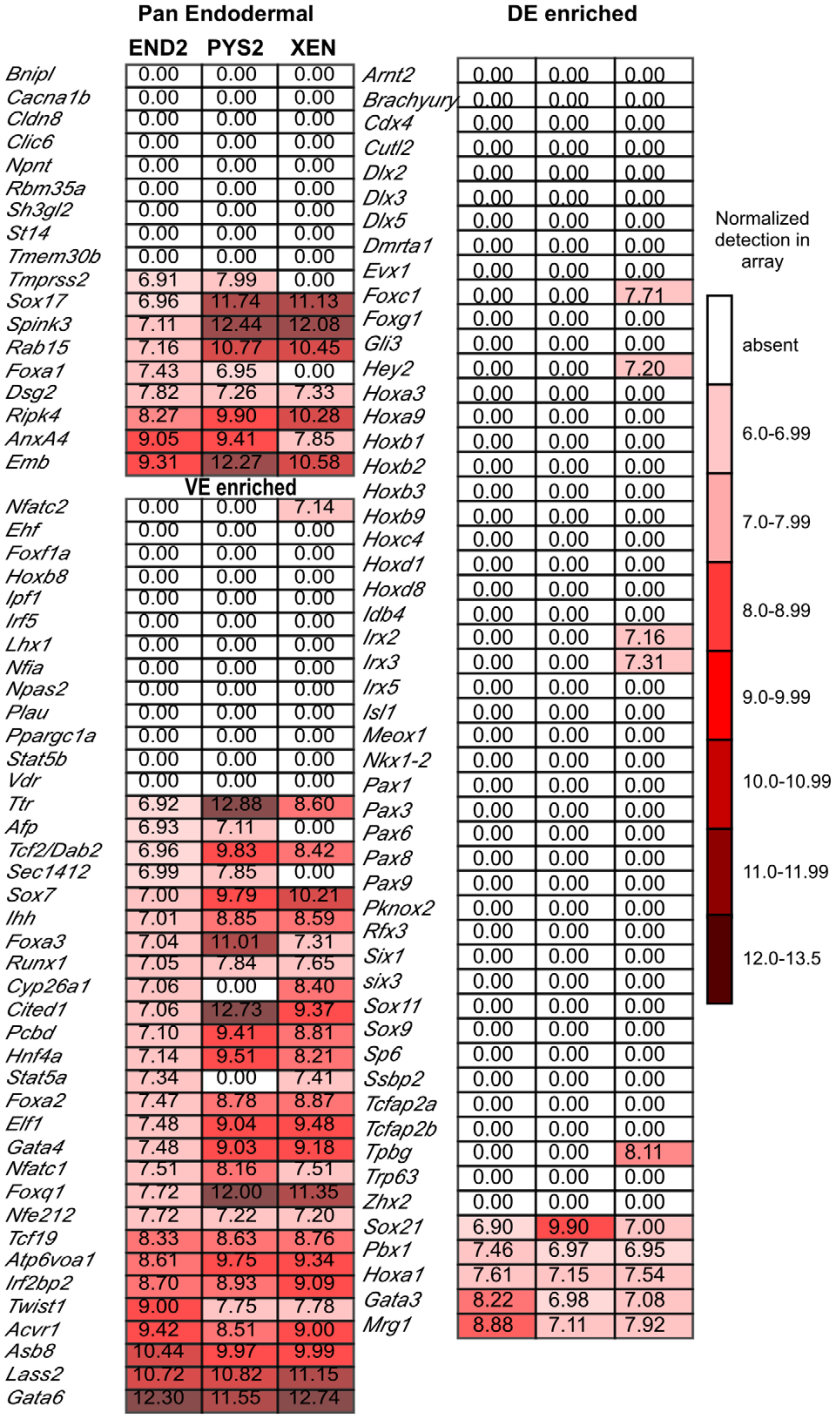
Heat map representation of detailed marker analysis. Microarray data of markers distinguishing VE from DE according to Sherwood *et al*. [2]. Only a subset of the previously examined pan endodermal markers are expressed in the array. This refines the list of true pan endodermal markers to include *Sox17, Spink3, Rab15, Dsg2, Ripk4, AnxA4* and *Emb*. “VE enriched” genes indicate factors that were found to be expressed in VE plus other lineages but not DE. Our analysis suggests that a subset of these factors may not be present in all VE subtypes and thus may represent regionalized VE markers. “DE enriched” represents genes found, in Sherwood *et al.* [2], to be expressed in DE and other subtypes but not in VE. These data strongly suggest that the ExEn cell lines are not similar to DE. Fluorescence data was marked as 0.00 if the p-value of detection was greater than 0.01.

The microarray data were then examined for expression of a large panel of markers identified in Sherwood *et al.* that are described as distinguishing between DE and VE (Fig. 5). Many, but not all, of the pan-endodermal markers are expressed by these cell lines [9/18 (50%), END2, PYS2 and 7/18 (39%), XEN cells]. These data indicate that a subset of these markers are not truly pan endodermal but add further support to the characterization of *Sox17, Spink3 Rab 15, Dsg2, Ripk4, AnxA4* and *Emb* as true pan endodermal markers. In addition, markers that were described as VE-enriched are highly expressed in the ExEn cell lines (65%, END2, 60% PYS2 and 62.5% XEN). By contrast, ExEn cells express only a small percentage of markers that distinguish DE from VE (9.6% END2, PYS2, 19% XEN cells). It should be noted, however that these markers are not exclusive to the DE and VE, and thus their expression in ExEn cell lines may indicate the presence of other lineages such as parietal endoderm.

**Figure 6:**
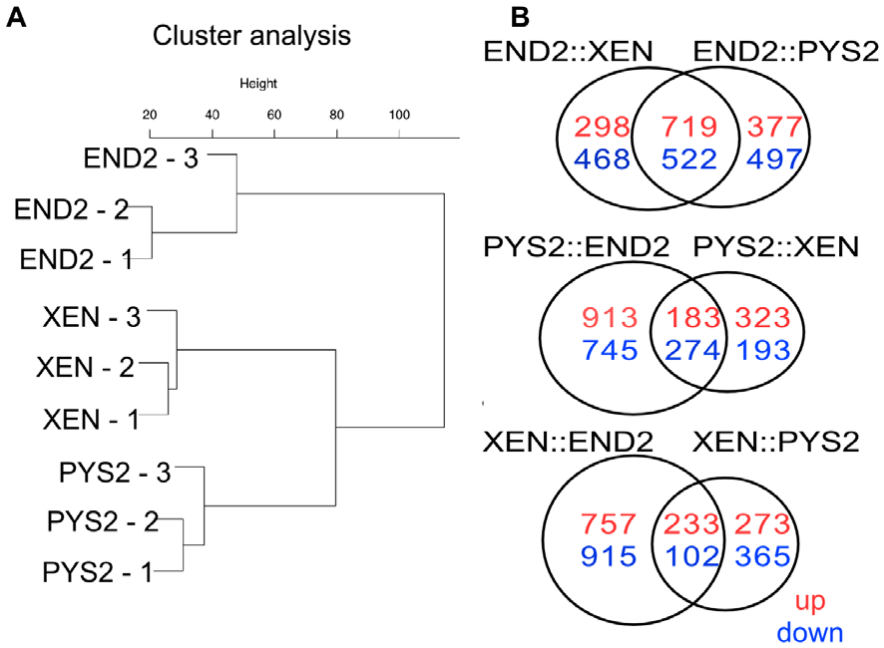
Cluster analysis of microarray data. **A.** Cluster Dendrogram representing the amount of variance in markers commonly expressed through microarray analysis by each of the ExEn cell lines (in triplicates). **B.** Venn Diagrams representing the number of genes in the array that are either upregulated (red) or downregulated (blue) in pair-wise comparisons between the different cell lines. Probes are called as “present” if the p-value for detection was less than 0.01.

Altogether these findings suggest that all of the cell lines examined in these studies are similar to extra-embryonic endodermal lineages and probably represent mixed populations of PrE derivatives. In addition, microarray analysis reveals distinct differences between these cell lines. To further understand these differences, cluster analysis (Fig. 6A) was performed. This supported the existence of significant molecular differences between the three ExEn cell lines. The greatest overall differences were found when END2 cells were compared to the other two cell lines (PYS2 and XEN). This finding is consistent with our previous analysis. We also compared the number of genes that are differentially expressed in pair-wise comparisons of the three cell lines, confirming our findings from the cluster analysis (Fig. 6B).

Therefore, while demonstrating that the three cell lines exhibit characteristics of the VE, these studies also highlight the fact that they do, nonetheless, have significant molecular differences between them. Since a subpopulation of the VE, the AVE has been shown to have heart-inducing ability, it is possible that these molecular differences might also reflect differences in the ability of these cell lines to activate and/or enhance cardiac differentiation in ES cells. Indeed both END2 and PYS2 cells have already been shown to possess heart inducing ability [4], [5], [33], [34], [35].

### Microarray analysis

To examine the microarray data in more detail, we performed pathway and tissue expression analysis on a total of 6094 annotated gene IDs that were detected as present in at least one of the three cell lines (based on a p-value of detection <0.01) using the Database for Annotation, Visualization and Integrated Discovery (DAVID) [36], [37]. Tissue expression analysis revealed that the top non-cancer tissue hit for this list of gene IDs was for liver (with a p-value of 5.3E-115), which is expected given the high degree of overlap between markers for the liver and the VE.

**Figure 7:**
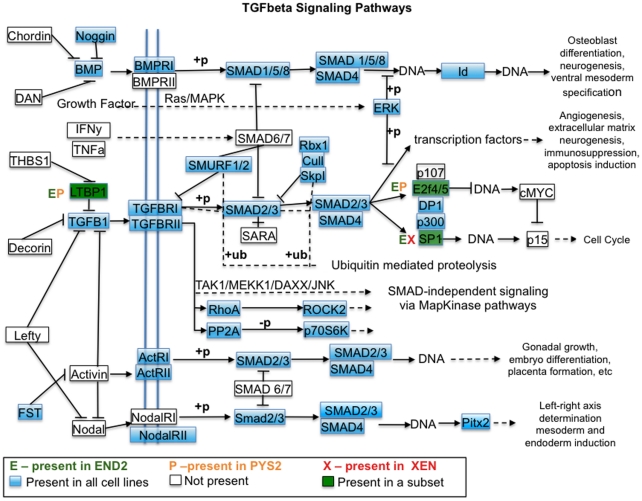
TGFbeta signaling pathways are active in heart inducing cell lines. Diagram of TGFbeta signaling pathways from the Kyoto Encyclopedia of Genes and Genomes [75], [76], [77] showing pathway components that are considered to be present based on a p-value of detection <0.01. Factors indicated in blue are present in all of the ExEn cell lines. Factors present in green are present in a subset of the cell lines (the particular cell lines are indicated by letters adjacent to the box indicating the factor).

**Figure 8:**
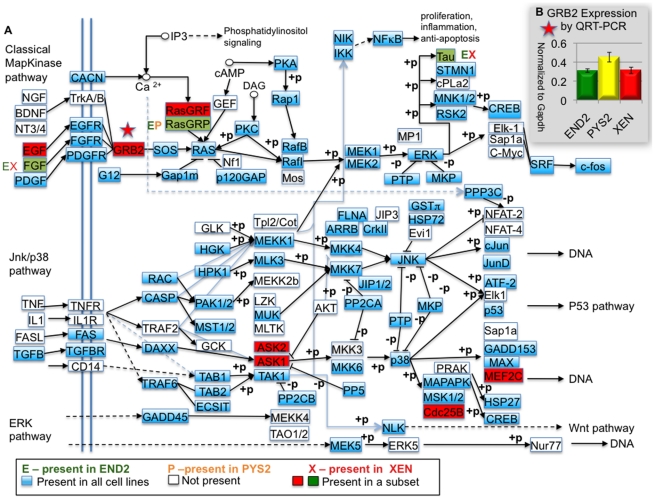
Heart inducing endoderm signals through the JNK/MAP Kinase and the TAK1/NLK pathways. **A.** Diagram of MAP Kinase signaling pathways after the Kyoto Encyclopedia of Genes and Genomes [75], [76], [77] showing pathway components that are considered present based on a p-value of detection <0.01. Factors indicated in blue are present in all of the ExEn cell lines. Factors highlighted in green are present in a subset of the cell lines (the particular cell lines are indicated by letters adjacent to the box indicating the factor). Factors indicated in red are present in XEN cells only. **B.** qRT-PCR data showing expression of Grb2 in END2 (green), PYS2 (yellow) and XEN cell (red) respectively.

To determine the signaling pathways that characterize these cell lines, the DAVID bioinformatics tool was used to compare the 6094 gene IDs present in the arrays to the BIOCARTA pathways database. This analysis revealed that the top pathways (not directly related to cell cycle) in the ExEn lines were the MAP Kinase (p-value, 2.7E-3) and TGFbeta (p-value, 3.1E-3) signaling pathways. Gene expression in these pathways was described by comparing our gene list to the TGFbeta and MAP Kinase pathways described in the Kyoto Encyclopedia for Genes and Genomes (Fig. 7 and Fig. 8). A detailed analysis of the TGFbeta pathway suggests that all three of these cell lines are capable of responding to all known subgroups of TGFbeta family members (Fig. 7). By comparison, an analysis of known MAP Kinase signaling pathways suggests that only XEN cells have a fully intact classical MAP Kinase signaling pathway since it was the only cell line in which *Grb2* is present by microarray (Fig. 8A). By contrast microarray data suggests that END2 and PYS2 cells likely signal through the TAK1/p38/JNK and the TAK1/NLK pathways. Although we found a better than 80% correlation between microarray and qRT-PCR data, we decided to confirm the presence or absence of *Grb2* expression in these cell lines by qRT-PCR (Fig. 8B). By PCR, we found that each of the three cell lines expresses mRNA for *Grb2* at similar levels. Taken together these analyses suggest that each of the three endodermal cell lines can signal through both classical and non-classical MAP Kinase signaling pathways.

In addition, we have previously shown [40] that signaling of TGFbeta family members in the endoderm is required for heart development. Together these pathway analyses (Fig. 7, 8) reveal that the effects of TGFbeta signaling could be mediated by either traditional SMAD-dependent signaling or by a SMAD independent pathway involving either TAK1/p38/JNK or TAK1/NLK.

**Figure 9:**
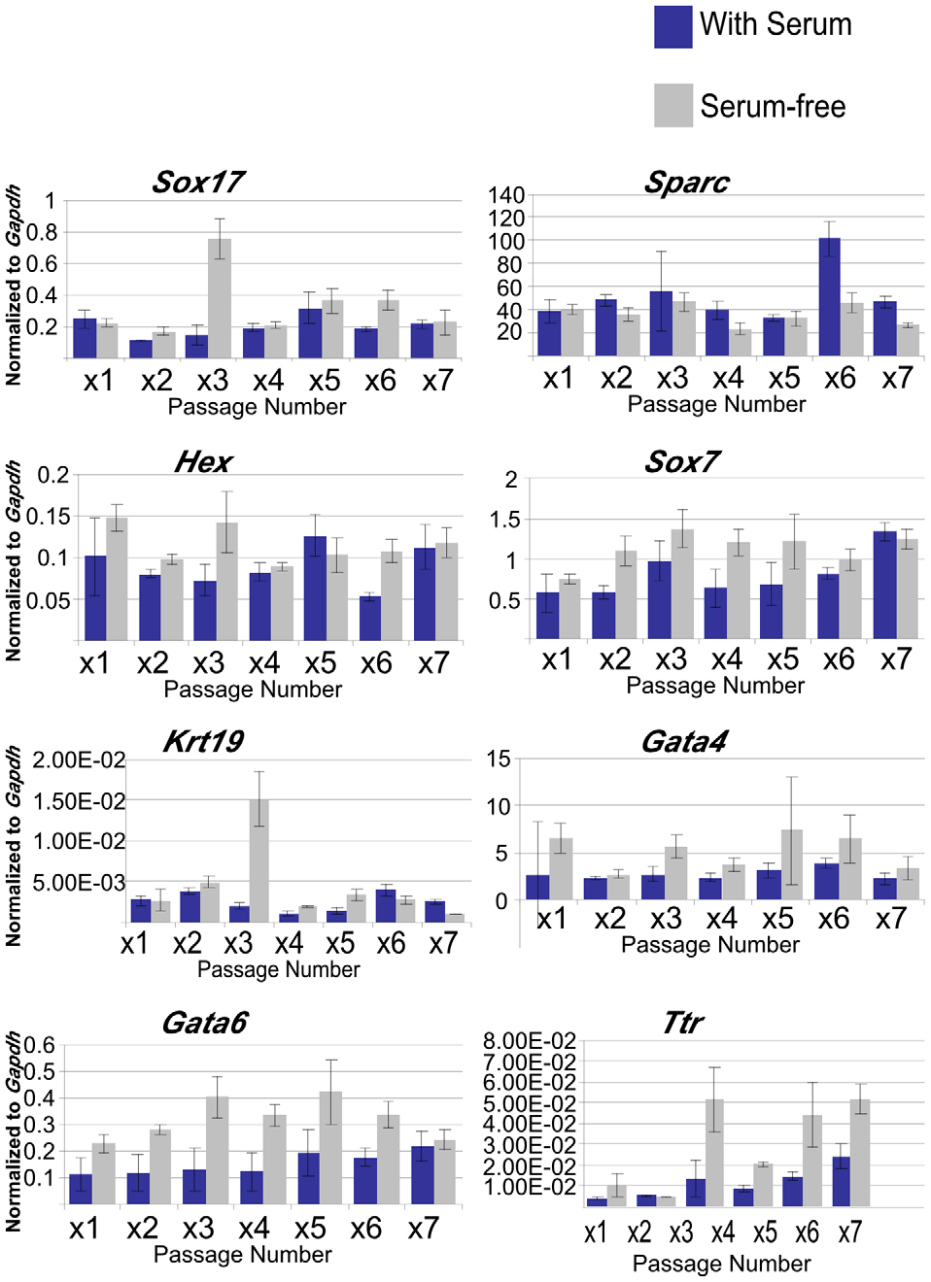
Marker expression in XEN cells is stable over several passages when cells are grown under standard serum-containing medium. qRT-PCR data comparing marker expression in XEN cells grown under standard serum-containing conditions to XEN cells grown in serum-free medium. Despite some spikes in marker expression at some passages, over the entire course of this experiment there were no obvious trends (either upward or downward changes in expression) in any of the markers assessed. In addition, while some markers were differentially expressed between the two culture conditions, there was also no obvious change in marker expression in the serum-free medium when assessed over the entire course of the experiment. This suggests that XEN cells are stable when grown in culture over several passages.

Finally, since END2 and PYS2 cells have been shown to secrete factors that induce differentiation in ES cells it is likely that XEN cells will have a similar inductive ability. Before this can be established, however, it is important to show that XEN cells are stable when maintained in culture. To address this question, we collected XEN cells at 70% confluence to ensure that cell density is equivalent for all analyses over seven passages. We then assessed the expression of a number of ExEn markers at each passage. In parallel, XEN cells were passaged in a serum-free medium to determine if gene expression in these cells is sensitive to culture conditions (Fig. 9). We found that despite spikes in some markers at certain passages, that cells grown in standard serum-containing medium are quite stable over seven passages and that there were no obvious trends in marker expression during the duration of this experiment. This suggests that XEN cells are stable in standard medium for at least short-term culture. When XEN cells are grown in serum-free medium we again noticed spikes in expression of some markers at some passages but again found no obvious trends in marker expression. We did however note that overall expression of *Gata6* and *Ttr* was different between the two culture conditions whereas other markers were expressed at statistically the same levels at most passages.

## Discussion

The endoderm makes up only a small percentage of cells of the early embryo. Cells within the endoderm layers of the early embryo exist in simple cuboidal or squamous epithelia, making them difficult to isolate mechanically from embryos. Because of these challenges to the embryological study of endodermal cells in the mouse embryo, many recent studies have relied on the use of endodermal-like cell lines that can mimic the functions of the early endoderm. For example END2 cells have been shown to enhance myocardial differentiation of both human and mouse ES cells [4], [33], [34] and to mimic the effect of the VE in activating cardiac formation from the undifferentiated mesoderm from the mouse embryo [35]. While it is clear from these studies that END2 cells mimic the effects of the AVE, the fact that they were originally derived from ECs raises some doubt as to whether they perfectly recapitulate the endogenous signals secreted by the AVE.

An added complication is the significant overlap between genes that mark the ExEn and those that mark the DE. This makes the analysis of *in vitro* differentiation of endodermal cell types difficult and thus poses a major hurdle for attempts to derive endodermal cell types for ES or other sources that might be used for therapeutic purposes.

Our studies address both of these concerns. First, we have undertaken an in depth analysis of the newly characterized XEN stem cells [6] which like the EC-derived END2 and PYS2 cells, express markers for the AVE. XEN cells can thus can be used to study the inductive effects that have been attributed to the AVE, including heart formation, primitive streak initiation and forebrain induction. In addition, since XEN cells are derived from mouse blastocysts, it will be possible to derive XEN cells from mice with deletions of genes thought to be involved in AVE function thereby providing an assay to directly test their function in the primitive endoderm. Finally, by comparing the three related but dissimilar cell lines, we are able to refine the list of markers that distinguish between different subtypes of endoderm including those that distinguish DE from VE.

### Inductive and morphogenetic functions of the extra-embryonic endoderm

Endoderm both embryonic and extra-embryonic has been shown in various model systems to play an inductive role in the differentiation of mesodermal and ectodermal tissues that adjoin it. The VE in particular has been proposed to play important roles in heart formation, primitive streak formation and the development of the forebrain.

Studies in amphibian embryos dating back to the 1960s demonstrate that signals from the pregastrula endoderm support myocardial differentiation [38], [39], [40], [41], [42], [43], [44], [45]. This finding is supported by studies that the AVE of the mouse [46] and the hypoblast of avian embryos [47], [48], [49], also support cardiac differentiation. In addition, endoderm isolated from avian embryos has been shown to enhance cardiac differentiation when co-cultured with mouse ES cells [50]. Importantly, these studies indicate that cardiac specification requires a VE signal only transiently, from early to mid-gastrulation [47], [51], [52] and that signals from the DE are required only later, for proliferation of cardiomyocytes and the initiation of beating [53]. Thus it will be of particular importance to separate the molecular signals of the DE from the VE in order to uncover the specific signals that mediate endoderm's ability to activate and later support myocardial differentiation.

The VE has also been implicated as providing signals required for streak elongation. This hypothesis has come largely from the observation of an anterior migration of the AVE just prior to the onset of gastrulation [54] and studies in avian embryos showing that rotation of the hypoblast (which is equivalent to the AVE [55]) results in a repositioning of the primitive streak [55], [56], [57]. A more in depth molecular analysis of this process has revealed greater complexity than was previously anticipated. First, removal of the hypoblast does not eliminate streak formation but rather results in the formation of multiple streaks. This suggests that the hypoblast does not activate streak elongation but rather acts to limit streak formation to a single location [58]. Simultaneously, FGF signaling from the hypoblast activates localized expression of genes associated with the establishment of planar cell polarity and defines the site of active streak elongation [59].

Finally, two specific observations lead to the hypothesis that AVE might serve as an inducing population for the vertebrate forebrain. First, ablation of the AVE of the mouse leads to a loss of *Hesx* expression in the anterior neural folds of the mouse (although other markers assessed were normal) [60]. Second, chimeric analysis of mouse embryos possessing homozygous deletion of *Otx2* [61], [62] and *Lim1* [63] or that are double mutant for *Lim1* and *HNF3*β [64], suggest that AVE expression of these genes is necessary for normal axis and forebrain development. Together these data suggest that signaling from the AVE is necessary for normal forebrain development. However, the AVE does not directly activate forebrain differentiation. Grafting of the rabbit AVE [65] or chick hypoblast to naïve epiblast that is capable of forming neural tissue results in the transient ectopic expression of neural markers but these markers are not maintained and host tissues do not form neural plate structures [55]. Similarly, explant co-cultures of AVE and mouse epiblast do not induce the expression of anterior neural markers but instead suppress posterior neural differentiation [66]. So what is the mechanism by which VE helps to pattern the forebrain if it does not act as a forebrain inducer? 1) Transient activation of early neural markers suggests that the VE may prime the ectoderm for neural development, 2) The AVE appears to repress posterior development and 3) it appears that the VE directs morphogenetic movements in the ectoderm and mesoderm that are required for normal axis formation [reviewed in: [55], [66]. This hypothesis is consistent with the hypoblast rotation experiments described in the previous section.

Together, these studies highlight the importance of the VE generally and the AVE specifically in the early patterning of the embryo and subsequent organ formation.

### Does the extra-embryonic endoderm make cellular contributions to endodermal organ formation?

Until recently, the dogma of endoderm formation in the mouse has asserted that the visceral endoderm that surrounds the embryonic epiblast prior to gastrulation is actively displaced by the forming definitive endoderm and contributes only to extra-embryonic structures [67]. This conclusion was based largely on the observation of gene expression patterns showing markers such as *alpha-fetoprotein* being lost in the endodermal cells overlying the epiblast during gastrulation and low-resolution fate mapping studies. With the advent of multiple lineage labels and time-lapse live imaging, we have clearly demonstrated that all or most the visceral endoderm that overlies the pregastrula epiblast is integrated into the definitive endoderm rather than being displaced by it. Concomitantly, these cells lose expression of visceral endoderm markers and gain expression of markers for the definitive endoderm. This lineage study also demonstrates that VE-derived cells contribute to gut formation [27]. This shows that there are distinct regions of embryonic (EmVE) and extra-embryonic (ExVE) visceral endoderm with unique fates in the embryo and suggests that these EmVE cells may contribute to endodermal organ formation. While none of the ExEn cell lines that we assessed expressed markers for the definitive endoderm, it remains to be determined if these cells can be coaxed by the addition of growth factors to differentiate along definitive endoderm lineages and adopt fates associated with the gut or its associated organs. If this turns out to be the case, then in addition to being a useful tool for the study of the inductive properties of the ExEn, XEN cells might also serve a stem population from which to derive differentiated endodermal cell types.

### TAK1/p38/JNK/NLK pathways in these cells types

Above, we presented genetic and embryological data that the VE acts, in part, to direct cell movements in the embryo. Consistent with this, we found that each of the three ExEn cell lines possesses intact pathways for non-classical MAP Kinase signaling acting through JNK [68] and NLK [69]. Since both of these factors are associated with planar cell polarity, it supports the notion that these pathways may play a pivotal role in the endoderm's ability to direct morphogenetic movements. Indeed, JNK Kinase has been shown to be necessary for heart induction downstream of the non-canonical Wnt, Wnt11 [70]. In addition, TAK1, which is immediately upstream of NLK is essential for cardiac differentiation in P19 [71], and TAK1 [72] and TAB1[73] mutants show defects in cardiac morphogenesis. It remains to be determined whether these defects result from a specific endodermal requirement for these signals or arise from a more general requirement in embryonic tissues. Nonetheless these findings lend support to a model in which a non-classical MAP Kinase pathway mediated by TAK1/p38/JNK or TAK1/NLK mediate some functions of the endoderm.

### Conclusions

Endoderm, both embryonic and extra-embryonic plays important morphogenetic functions in the mouse and other vertebrate embryos. We are only just beginning to refine our understanding of the different types of endoderm and the molecular mechanisms by which they mediate their functions in development. Here, we undertake a thorough analysis of ExEn cell lines to help refine the list of markers that define various endodermal cell types. Specifically we provide further characterization of XEN cells [6] which may serve as a useful tool in the study of ExEn differentiation and function.

## Materials and Methods

### Cell Culture

XEN cells were derived from ICR strain blastocyst stage embryos according to standard procedures [6]. END2 cells were derived from P19 embryonal carcinoma cell lines [74] and PYS2 cells were derived from 129 strain mice tumor cells [1]. XEN and PYS2 cells were both maintained in high glucose Dulbecco's Modified Eagles Medium (DMEM) devoid of L-glutamine and sodium pyruvate (Mediatech). DMEM was enriched with 10% ES qualified Fetal Bovine Serum (GIBCO, lot: A15A00X), 1X nonessential amino acids (Mediatech), 1X L-glutamine, 1X sodium pyruvate (Mediatech) and β-mercaptoethanol (Sigma) was added to the medium to make a final concentration of 0.1 mM. Penicillin and streptomycin were then added in final concentrations of 100 units/ml and 100 µg/ml, respectively (Mediatech). This medium is referred to in the text as *standard medium*. END2 cells were grown in DMEM/F12 1∶1 media (Mediatech) supplemented with 10% FBS, L-glutamine, non-essential amino acids and penicillin/streptomycin (Mediatech). To test for the stability of these cells over several passages and to test their stability in different media, XEN cells were thawed and cultured as previously described. Cells were split onto two plates: one for standard (+ serum) and the other for serum-free culture. Serum-free medium is comprised of Knockout DMEM (Invitrogen), 10% Knockout SR (Invitogen), 1X nonessential amino acids (Mediatech), 1X L-glutamine, β-mercaptoethanol (Sigma) and penicillin/streptomycin (Mediatech). Cells were then collected at approximately 70% confluence after every passage for a total of 7 passages. RNA was isolated and cDNA was synthesized. qRT-PCR was performed with various endodermal markers and data analyzed for changes in marker expression over the 7 passages in serum containing and serum-free conditions.

### Real Time PCR

Cells were collected at 70% confluency, RNA was isolated using Tri Reagent (Sigma) and cDNA was transcribed using 1 µg RNA using Quantitect Reverse Transcription Kit (Qiagen). qRT-PCR reactions were carried out using a 1/20 dilution of template cDNA in SybrGreen Master Mix (Roche, cat #: 04707516001), on a Roche LightCycler ® 480 Real-Time PCR Instrument, and analyzed with the LightCycler 480 software package (version 1.5.0.39). Primers used in this study are as follows:

*Alpha-fetoprotein (Afp*): forward AGCTGACAACAAGG GGAGTG, reverse TTAATAATGGTTGTTGCCTGGA; *Cerberus-like (Cerl)*: forward GCAGACCTATGTGTGGA, reverse ATGAGACATGATCGCTTT; *Bmp2*: forward TGTGGGCCCTCATAAAGAAGC, reverse AGGGTGCAGGCAGGAAACATA; *Dkk-1*: forward TACAATGATGGCTCTCTGCAGCCT, reverse TGGTCAGAGGGCATGCATATTCCA; *Foxa2*: forward CGGCCAGCGAGTTAAAGTAT, reverse TCATGTTGCTCACGGAAGAG; *Gapdh*: forward AATGGATACGGCTACAGC, reverse GTGCAGCGAACTTTATTG; *Gata4*: forward CATCAAATCGCAGCCT, reverse AAGCAAGCTAGAGTCCT; *Gata6*: forward ACCATCACCCGACCTACTCG, reverse CGACAGGTCCTCCAACAGGT; *Grb2*: forward TTGTGTGTCCCAGTGTGCAA reverse AGCTCAGCTCATCGTCAGCA; *Hex*: forward GGAGGCTGATCTTGACT, reverse GTAGGGACTGCGTCAT; *Hnf4a*: forward CGAACAGATCCAGTTCATCAAG, reverse ATGTGTTCTTGCATCAGGTGAG; *Cytokeratin 19* (*Krt19)*: forward ATCCAGATAAGCAAGACCGAAGT, reverse ATCTGTGACAGCTGGACTCCATA; *Laminin B1*(*Lamb1)*: forward CAGAATGCAGACGATGTTAAGAA, reverse GGCATCTGCTGACTCTTCAGT; reverse AGCGTGTACCCTATTGG; *Platelet-derived growth factor alpha (Pdgfra)*: forward CCTCAGCGAGATAGTGGAGAAC, reverse ACCGATGTACGCATTATCAGAGT; *Sox17*: forward GGAATCCAACCAGCCCACTG, reverse GGACACCACGGAGGAAATGG; *Sox7*: forward CAAGGATGAGAGGAAACGTCTG, reverse TCATCCACATAGGGTCTCTTCTG; *Sparc*: forward AGGGCCTGGATCTTCTTTCTC, reverse CAAATTCTCCCATTTCCACCT; *transthyretin (Ttr)* forward TTCACAGCCAACGACTCTGG, reverse AATGCTTCAGGGCATCTTCC; *t-type plasminogen activator* (*tPA*): forward CTGACTGGACAGAGTGTGAGCTT, reverse ACAGAT GCT GTGAGGTGCAG; *urokinase-type Plasminogen activator* (*uPa)*: forward CAGCTCATCTTGCACGAATACTA, reverse AGATGGTCTGTATGGACCTGGAT; *Villin1(Vil1)*: forward TCAAGTGGAGTAACACCAAATCC, reverse CTAGTGAAGTCTTCGGTGGACAG.

### Immunohistochemistry

Cells were washed with PBS and fixed in 4% PFA 30 minutes, then blocked with 3% FBS-0.3% Triton in PBS. Primary antibodies were then added, and incubated overnight at 4°C. Cells were washed with PBS and blocked for 30 minutes at room temperature. Secondary antibodies were added and cells were incubated overnight at 4°C. Finally, cells were washed with PBS and cover slipped with Vectashield mounting medium containing DAPI.

### Scanning Electron Microscopy

Cells were passaged on to gelatin-coated plastic coverslips. A day later, they were rinsed once with PBS and fixed at room temperature in 2.5% Glutaraldehyde/2% PFA in 0.075M Cacodylate buffer pH 7.5 for one hour. They were then dehydrated in a graded ethanol series. Cells were then critical-point dried in a Denton JCP-1 Critical Point Drying Apparatus and subsequently coated with gold/palladium in a Denton Vacuum Desk 1V sputter coating system. Imaging was carried out with Zeiss Field Emission Supra 25 Scanning Electron microscope.

### Microarray Analysis

Total RNA was isolated with Qiagen RNeasy Mini Kit and used to probe Illumina expression array (MouseWG-6_V2_0_R0_11278593) in triplicate for each of three heart-inducing cell lines using Illumina BeadStudio version 3.4.0. The raw Illumina data (9 arrays) was analyzed using Bioconductor packages. The data was first normalized using LumiExpresso_( ) function. The differentially expressed genes in each pair-wise comparison were obtained using Limma_( ) R-package. For gene ontology studies, Illumina probes were mapped to gene symbol names using—getAnnote.Illumina— ("MouseWG-6_V2_0_R0_11278593_A.bz2") downloaded from Bioconductor website: http://www.bioconductor.org/download.

Pathway and expression analysis was carried out using DAVID Bioinformatics Resources 2008 sponsored by the National Institute of Allergy and Infectious Diseases (NIAID), NIH, at http://david.abcc.ncifcrf.gov/ [36], [37] and the Kyoto Encyclopedia of Genes and Genomes http://www.genome.jp/kegg/ [75], [76], [77]. This data is MIAME compliant and has been deposited in NCBI's Gene Expression Omnibus [78]. All data is accessible through GEO Series accession number GSE19564 (http://www.ncbi.nlm.nih.gov/geo/query/acc.cgi?acc=GSE1956).
